# Acute medical care resource utilization of frail older patients presenting at the emergency department

**DOI:** 10.1007/s41999-026-01503-0

**Published:** 2026-05-26

**Authors:** Linda Becude, Frouke M. Engelaer, Leonie J. van Meer, M. Christien van der Linden, Merel van Loon-van Gaalen, Saskia L. M. A. Beeres, Douwe H. Biesma, Gerard J. Blauw, Frederiek van den Bos

**Affiliations:** 1 Department of Acute Internal Medicine, Leiden University Medical Center, Leiden, The Netherlands; 2 Department of Geriatric Medicine, Haaglanden Medical Center, The Hague, The Netherlands; 3 Department of Geriatric Medicine, Leiden University Medical Center, Leiden, The Netherlands; 4 Department of Emergency Medicine, Haaglanden Medical Center, The Hague, The Netherlands; 5 Department of Cardiology, Leiden University Medical Center, Leiden, The Netherlands; 6 Department of Internal Medicine, Leiden University Medical Center, Leiden, The Netherlands

**Keywords:** Frail patients, Geriatric, Medical care resource utilization, Alternative for hospitalization, Appropriate acute care use, Emergency department

## Abstract

**Purpose:**

The emergency department (ED) has changed significantly due to technical innovations, centralization of care and further medical specialization. It is critical to assess whether the ED remains the most appropriate setting for older (frail) patients, given their multimorbidity, differing life goals, and the need to maintain acute care accessible.

**Methods:**

This study assesses ED visits, focusing on differences in acute medical care resource utilization between younger and older patients, including the impact of age and frailty. A retrospective case series was performed over nearly six years, using descriptive trend analysis and multivariable regression.

**Results:**

This study included 163,140 patients, accounting for 274,462 ED visits. Median age was 46 years (IQR 25–66), and 48.5% were female. Patients aged ≥ 70 years (21.5% of visits) demonstrated higher use of acute care resources, including laboratory tests (OR 1.49), X-rays (OR 1.97), CT scans (OR 1.78), ambulance transport (OR 2.98), specialist consultations (OR 1.80), and hospital admissions (OR 2.83) compared to younger patients. Frail older patients had even greater utilization, independent of age, with adjusted ORs of 2.54 for ambulance transport and 4.45 for consultations.

**Conclusion:**

Older, especially frail, patients utilize more acute medical care resources and are more frequently hospitalized than younger individuals. These findings support the need for holistic, transmural care models that better align with patients’ needs and reduce potentially non-beneficial interventions. The current ED setting may not be optimal for delivering such care. Tailored care pathways and alternatives to conventional hospitalization may improve patient outcomes and healthcare sustainability.

## Key summary points

**Aim:**

What is the trend in use of acute medical care resource utilization and hospital admissions among older, particularly frail, patients compared to younger patients.

**Findings:**

Older patients presenting on the emergency department (ED) have high utilization of acute medical care resources, such as transport by ambulance, diagnostic procedures (laboratory or radiology), length of ED stay, admissions to the hospital and revisits compared to younger patients.

The higher rates of acute medical care utilization are particularly pronounced in frail older adults, independent of age, and specifically when focusing on use of ambulance services and consultations at the ED.

**Message:**

To improve outcomes and reduce nonbeneficial care for frail older adults, healthcare systems should prioritize shared decision making, advance care planning, and the development of integrated hospital–primary care collaborations as sustainable alternatives to conventional hospitalization.

## Introduction

The acute healthcare chain in the Netherlands has undergone substantial transformations in the recent years. Concentration and centralization of acute care alongside technical innovations culminated in an extremely complex emergency department (ED) [1, 2]. Further advancements, such as the establishment of general practitioner’s posts at the ED, acute admission departments, the presence of ED physicians and medical specialists working within the ED. Combined with the integration of technological innovations, this contributes to increased sub specialization, and hence more fragmentation of medical professionals and care within the ED [3, 4]. These changes improved the quality of acute care for most individuals, presenting with an acute medical need.

However, the ED is less well equipped for frail older patients with multimorbidity. They need a more holistic approach, whereas the sub specialized ED is focusing on single medical conditions. Even when frail older patients are in urgent vital needs, traditional life-saving measures and general protocols should not unquestioningly be followed, as they may not be beneficial for these patients and may not align with their wishes [5]. This misalignment may lead to disproportional and potentially harmful diagnostic measures, incidental findings and unnecessary invasive diagnostics. For part of these frail older patients, the mentioned harm could be preventable, as part of this group is not in need of acute ED medical care, but mainly in need of other care questions, like extra home or nursing care that is not readily available in their home situation [6].

This prompts the question of whether the specialized ED is the best setting for frail older patients with multimorbidity in acute or less-urgent care needs. Owing to the ageing populating and shortages of staff, this question is of upmost importance, but literature has not yet addressed this. Addressing this question requires first a thorough examination of the unique patterns and characteristics of older patients in the ED. Therefore, we aim to provide insight into the trends of admissions of older patients to a teaching hospital with one of the largest EDs in the Netherlands using a descriptive study. Furthermore, we aim to evaluate how the use of acute medical care resource of frail older patients differs from other ED patients.

## Methods

### Study design and outcome

A retrospective case series analysis was performed between 1 January 2019 to 1 October 2024, at Haaglanden Medical Centre (HMC) location Westeinde, located in The Hague, the Netherlands. It concerns a descriptive trend analysis in which we compared the subset of frail older patient in the ED with other ED patients in terms of demographic data, main diagnosis, medical care utilization and destination after the ED visit. The study was conducted in accordance with the principles of the Declaration of Helsinki and was approved by the departmental research committee and the institutional non-WMO committee. As the Medical Research Involving Human Subjects Act does not apply to this study, formal medical ethics committee approval was not required.

### Setting

The HMC is a teaching hospital, with 52,000 ED visits (adults and children) annually. The ED comprises 24 beds and functions as a regional trauma center. The hospital has a total of 300 staffed ward beds and 16 Intensive Care Unit beds. Triage is performed according to the Manchester Triage System [7]. Patients suitable for assessment by a general practitioner (GP) are redirected to the GP cooperative (GPC). Critical patients are assigned to an ED room, while those with acuity levels of 3–5 wait in the waiting room if beds are unavailable.

### Patients

All consecutive patients who presented at the ED in this period were included. Patients who were electively treated on the Cardiology Emergency Department, such as those scheduled for cardioversion, were excluded. Patients for whom no specialist was assigned and test patients were also excluded. Patients were categorized based on age into three groups: 0–17 years, 18–69 years, and 70 years and older. In this study we compared the patients of 70 years and older to the younger adults.

**Fig. 1:**
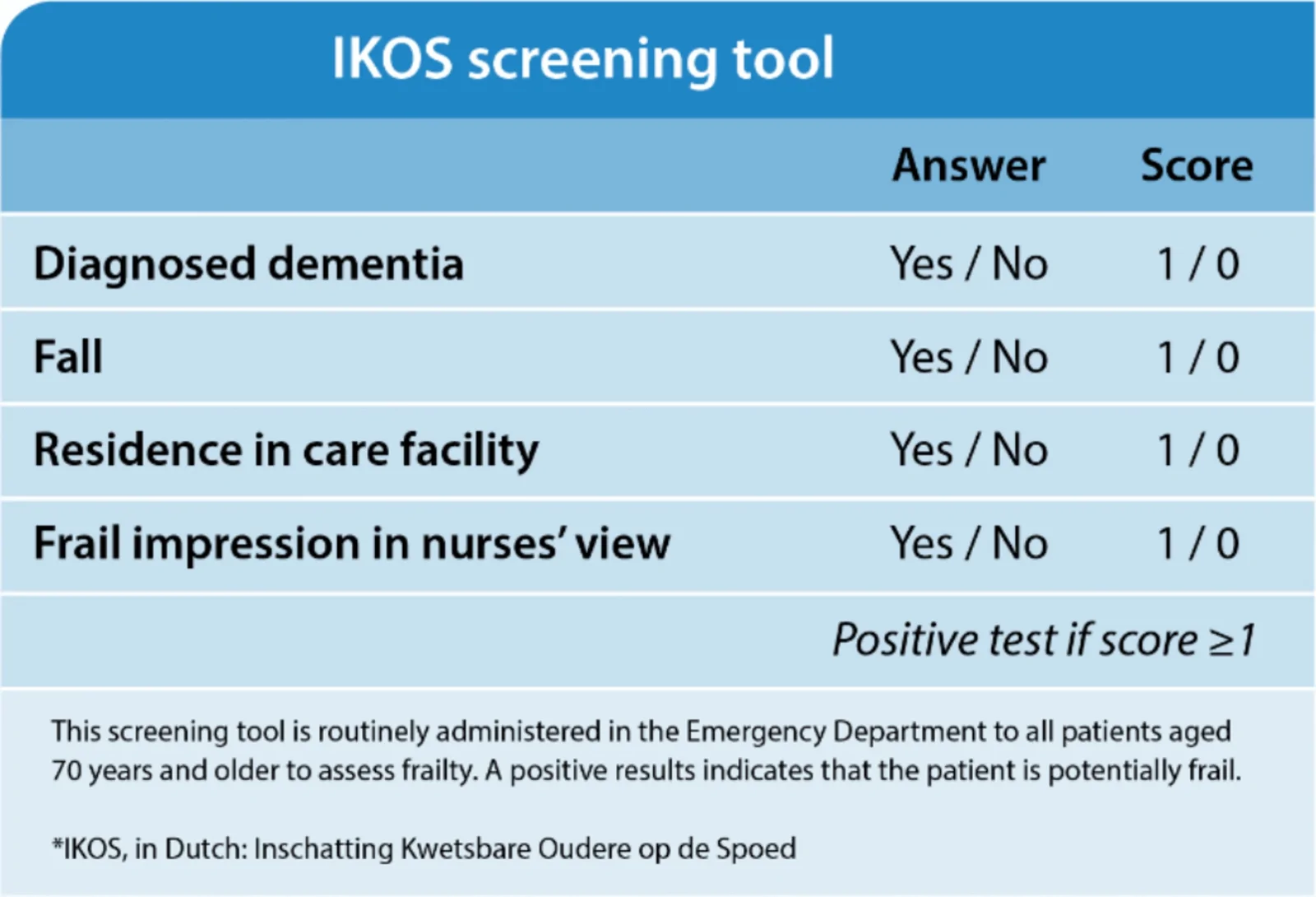
IKOS (In Dutch: Inschatting Kwetsbare Oudere op de Spoed) screening tool

Starting from November 11th, 2022, a screening tool (IKOS, in Dutch: Inschatting Kwetsbare Oudere op de Spoed) to assess frailty was introduced for all patients aged 70 years and older. This tool comprises four questions completed by the nurse (Fig. 1). The questions assess whether the patients has experienced a fall, has a diagnosed dementia, resides in a care facility and finally a clinical assessment whether or not the patient gives a frail impression in the nurses estimation. If any of these questions are answered positively, the patient is considered potentially frail. These patients will, during the day and evening, receive a Geriatric Assessment (GA) adjusted to the acute care setting by an internist specialized in geriatric medicine (See Appendix 1 for the components of this GA). Within this subgroup of patients aged 70 years and older, an additional comparison was performed in this study between those identified as potentially frail and those not considered frail based on the IKOS results.

### Data collection

All study data were obtained from the electronic medical record (EMR). The dataset was extracted in a structured and automated manner by the hospital’s Business Intelligence department; no manual data collection was performed. Prior to extraction, a predefined set of variables was specified by the research team, based on clinical relevance and technical availability within the EMR system. Collected variables included: demographic data; if the patient came by ambulance or private transportation; the results of the identification tool for frailty; main diagnosis; involved medical specialties; destination after ED visit; triage-urgency categories (defined by the maximum time to assessment: non-urgent, urgent, standard, very urgent, immediate) according to the Manchester Triage System, assigned by trained clinical staff; length of stay at the ED; length of hospital stay in case of admission; amount and type of radiology investigations and if blood tests were performed. The collected data was coded, and the amount of missing data was checked. Duplicated records, test patients and impossible correct data (negative values for length of stay in the ED or admission duration) were removed. Outliers were evaluated.

### Statistical analysis

Nominal data were presented as numbers and percentages. Continuous variables were described using means and standard deviation, or medians and interquartile ranges in cases of non-normal distribution. Differences in continuous variables between the frail older patients’ group and the control group were tested with *t* tests or Mann–Whitney U tests, depending on the distribution. Categorical variable differences between the groups were examined with Chi-square test. To evaluate whether the risk of utilizing more acute medical care resources–specifically for ambulance transport and consultations—was dependent of age or frailty, multivariable logistic regression analyses were conducted. Statistical analyses were performed using the Statistical Package for the Social Sciences (IBM Corp., IBM SPSS Statistics for Windows, version 28.0.1.0 Armonk, New York, USA).

## Results

### Study population

**Fig. 2:**
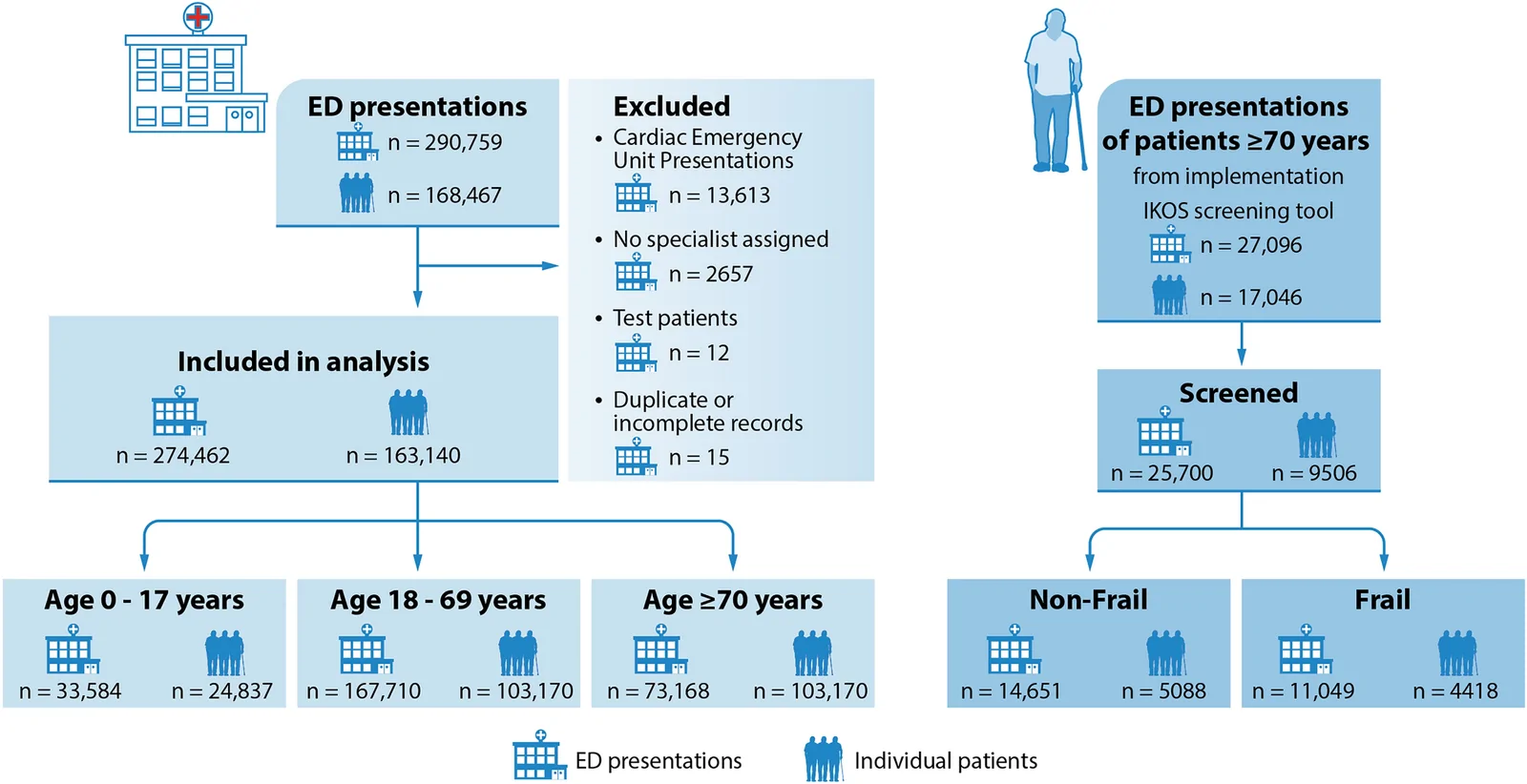
**a** Flowchart of patient inclusions of all patients presented on the emergency department (ED) between 1 January 1 2019 to 1 October 1 2024, **b** Flowchart of patient inclusions from the sub group of patients of ≥ 70 years presented from implementation of the frailty screening tool (IKOS)

During the 5-year and 9-month study period, 290,759 ED presentations were registered, with 16,297 visits being excluded mainly because of their electively presentation on the Cardiac Emergency Department. The remaining 274,462 ED presentations represented 163,140 individual patients, reflecting that some patients had multiple ED visits during the study period (Fig. 2a).

The baseline characteristics of the study population are presented in Table 1. Median age was 46 years (IQR 25–66). Among these patients, 24,937 were aged 0–17 year (15.2%), 103,170 (63.2%) were aged 18–69 year and 35,133 (21.5%) were 70 years and older. In total, 79,174 (48.5%) patients were female. The majority of patient presentations were for surgery/traumatology (37.1%) or internal medicine (25.6%). The most common presenting complaints included limb problems (14.7%), abdominal pain (12.0%) and feeling unwell (10.5%).

**Table 1:** Baseline demographics and clinical characteristics of the study population

Total number of patients *n*	163,140
Female *n (%)*	79,174 (48.5)
Median age in years^d^ [IQR]	46 [25–66]
Age in years^d^* n (%)*	
0—17 years	24,837 (15.2)
18—69 years	103,170 (63.2)
≥ 70 years	35,133 (21.5)
Born in the Netherlands^a^ *n (%)*	78,745 (48.3)
Total number of ED presentations ***n***	274,462
Acuity level^b,e^* n (%)*	
Non-urgent	1954 (0.8)
Standard	60,386 (23.3)
Urgent	136,795 (52.7)
Very urgent	56,594 (21.8)
Immediate	3756 (1.4)
Main specialism *n (%)*	
Surgery/traumatology	101,714 (37.1)
Internal medicine^f^	70,203 (25.6)
Neurology	34,122 (12.4)
Cardiology	29,340 (10.7)
Paediatrics	13,327 (4.9)
Other^g^	25,756 (9.3)
Presenting main problem^*c*^	
Limb problems	40,320 (16.2)
Abdominal pain	33,228 (13.3)
Unwell patient	28,881 (11.6)
Shortness of breath	23,406 (9.4)
Wounds and local infections	17,635 (7.1)
Falls	16,427 (6.6)
Chest pain and collapse	16,148 (6.5)
Headache	12,594 (5.1)
Urological problems	6907 (2.8)
Severe trauma	5798 (2.3)
Head injury	5107 (2.1)
Intoxications/exposure to toxic substances	5011 (2.0)
Other presenting problem^h^	37,482 (15.1)

^a^Based on 133,825 patients, due to 29,315 (17.9%) missings

^b^Based on 259,485 presentations, due to 14,977 (5.5%) missings

^c^Based on 248,944 presentations, due to 25,518 (9.3%) missings

^d^Based on data of first presentation on ED during study period is used

^e^Acuity based on Manchester Triage Scale levels

^f^Internal medicine: internal medicine, pulmonology and gastroenterology

^g^Other specialism: all visits for other specialism besides internal medicine, surgery/traumatology, neurology, cardiology, and paediatrics

^h^Other presenting problem: presenting problem occurring less than 5000 times during the study periods: allergy, asthma, diabetes, facial or dental problems, gastro intestinal problems, ear/nose/throat/eye problems, irritable/crying child, epileptic insult, neck pain, psychiatric disorders, sexually acquired infections

*ED* emergency department, *IQR* interquartile range

After the implementation of the IKOS frailty identification tool, there were 27,096 ED presentations of patients 70 years and older over a period of 1 year and 11.5 months. This cohort represents a specific subgroup within the overall study population. Of these, the IKOS score was completed for 25,700 (94.8%) presentations, with a positive result in 14,651 (57.0%) of the screened patients. (Fig. 2b).

### Utilization of acute care services per age category

Patients aged 70 years and above had significantly more acute care resource utilization compared to patients between 18 and 69 years old (Table 2). They had more laboratory investigations (44.2% vs 34.8%, OR 1.49 (95% CI 1.46–1.51)), X-Ray’s (40.5% vs 25.7%, OR 1.97 (95% CI 1.93–2.00)), and CT scans (32.6% vs 21.4%, OR 1.78 (95% CI 1.74–1.81)). They were more often transported by ambulance (57.4% vs 30.9%, OR 2.98 (95% CI 2.92–3.03)) and were more frequently admitted to the hospital (46% vs 23.2%, OR 2.82 (95% CI 2.78–2.88)). Furthermore, patients aged 70 years and above had more consultations in the ED of other specialists (24.1% vs 15.0%, OR 1.80 (95% CI 1.76–1.84)), had a longer length of stay at the ED (209 vs 163 min, *p* = < 0,05), and had more ED revisits within 30 days (14.2% vs 13.6%, OR 1.05 (95% CI (1.02–1.08)) as compared to patients between 18 and 69 years old. Only ultrasound use was found to be less used in patients aged 70 years and above (6.3% vs 10.4%, OR 0.58 (95% CI 0.56–0.60)).

**Table 2:** Medical care utility per age category for every ED presentation

	0—17 years (*n* = 33,584)	18—69 years (*n* = 167,710)	≥ 70 years (*n* = 73,168)	OR (95% CI)^d^
Transport^a^* n (%)*				
Ambulance	2773 (8.3)	51,470 (30.9)	41,604 (57.4)	2.98 (2.92–3.03)*
Private transport	30,104 (90.3)	111,395 (66.9)	28,495 (39.3)	0.32 (0.32–0.33)*
Other^e^	470 (1.4)	3639 (2.2)	2396 (3.3)	1.53 (1.45–1.61)*
Acuity level^f^b,*n (%)*				
Non-urgent	24,790 (80.7)	124,972 (78.5)	49,373 (70.9)	0.67 (0.65–0.68)*
Urgent	5917 (19.3)	34,198 (21.5)	20,235 (29.1)	1.50 (1.47–1.53)*
Diagnostics *n (%)*				
Laboratory	3456 (10.3)	58,339 (34.8)	32,346 (44.2)	1.49 (1.46–1.51)*
X-Ray	8029 (23.9)	42,128 (25.7)	29,618 (40.5)	1.97 (1.93–2.00)*
Ultrasound	2286 (6.8)	17,382 (10.4)	4602 (6.3)	0.58 (0.56–0.60)*
CT-scan	1110 (3.3)	35,859 (21.4)	23,846 (32.6)	1.78 (1.74–1.81)*
Destiny after ED visit^c^*n (%)*				
Admission	5035 (15.0)	38,717 (23.2)	33,567 (46.0)	2.83 (2.78–2.88)*
Home	24,958 (74.5)	111,373 (66.6)	32,312 (44.3)	0.40 (0.39–0.41)*
Outpatient clinic	2821 (8.4)	11,994 (7.2)	3337 (4.6)	0.62 (0.60–0.65)*
Institution	0 (0)	216 (0.1)	1517 (2.1)	16.41 (14.23–18.94)*
Mortuary	1 (0)	146 (0.1)	260 (0.4)	4.09 (3.34–5.01)*
Other^g^	680 (2.0)	4689 (2.8)	1927 (2.6)	0.94 (0.89–0.99)*
Number of patients who had at least one consultation of another specialist on the ED* n* (%)	4270 (12.7)	25,171 (15.0)	17,629 (24.1)	1.80 (1.76–1.84)*
Median length of stay (LOS) in ED in minutes [IQR]	114 [71–170]	163 [105–236]	209 [145–283]	*
ED revisit within 30 days *n* (%)	3007 (9.0)	22,851 (13.6)	10,383 (14.2)	1.05 (1.02–1.08)*

*CI* confidence interval, *ED* emergency department, *IQR* interquartile range, *LOS* length of stay, *OR* odds ratio

^*^All* p* values were statistically significant and were calculated using *χ*^2^ tests, except for median length of stay on ED, which was calculated using Mann–Whitney *U* test

^*a*^Based on 272,346 ED presentations, due to 2116 (0.8%) missings

^*b*^Based on 259,485 ED presentations, due to 14,977 (5.5%) missings

^*c*^Based on 273,550 ED presentations, due to 912 (0.3%) missings

^d^Comparison between patients of 18–69 years versus patients of ≥ 70 years

^e^Other transport: all transport modalities besides ambulance and private transport

^f^Acuity based on Manchester Triage Scale levels. Non-urgent: non-urgent and standard. Urgent: urgent, very urgent and immediate

^g^Other destiny after ED visit: all destiny’s after ED visits besides admission, home, outpatient clinic, institution and mortuary

### Utilization of acute care services for non-frail versus frail patients

Frail older patients were more often transported by ambulance (71.3% vs 45.9%, OR 2.89 (95% CI 2.75–3.05%)) compared to non-frail older patients (Table 3) The acuity level of frail patients was significantly more often assigned as non-urgent compared to non-frail patients (70.1% vs 68.7%, OR 1.22 (95% CI 1.16–1.29)). For most of the medical care utility outcomes, frail older patients received more diagnostics compared to non-frail older patients (laboratory investigations (78.6% vs 77.2%, OR 1.09 (95% CI 1.02–1.15)), X-Ray’s (51.8% vs 28.6%, OR 2.86 (95% CI 2.55–2.83)) and CT scans (38.8% vs 22.0%, OR 2.25 (95% CI 2.13–2.38))). Frail older patients also received more consultations (44.8% vs 14.8%, OR 4.73, (4.44–5.03)) and were more frequently admitted to the hospital (50.8% vs 39.5%, OR 1.58 (95% CI 1.50–1.66)). In addition, they had a longer length of stay at the ED (234 vs 200 min, *p* = < 0.05) and, in case of admission, a longer stay in the hospital (4.7 vs 2.7 days, *p* = < 0.05) in comparison with the less frail older patients.

**Table 3:** Baseline characteristics and medical care utility for patients of ≥70 years for non-frail and frail patient

	Non-Frail	Frail	OR (95% CI)
Total number of patients	4418	5088	
Female n (%)	2137 (48.4)	2962 (58.2)	
Median age in years [IQR]	76 [73–81]	81 [76–86]	
Total number of ED presentations	11,049	14,651	
Transport^a^* n (%)*			
Ambulance	4926 (45.9)	10,248 (71.3)	2.89 (2.75–3.05)*
Own transport	5804 (54.0)	4100 (28.5)	0.35 (0.33–0.37)*
Other^e^	9 (0.1)	15 (0.1)	1.26 (0.55–2.87)
Acuity level^b,f^* n (%)*			
Non-urgent	7173 (68.7)	10,162 (70.1)	1.22 (1.16–1.29)*
Urgent	3257 (31.2)	4329 (29.9)	1.00 (0.950–1.06)
Diagnostics *n (%)*			
Laboratory	8535 (77.2)	11,522 (78.6)	1.09 (1.02–1.15)*
X-Ray	3158 (28.6)	7588 (51.8)	2.68 (2.55–2.83)*
Ultrasound	774 (7.0)	806 (5.5)	0.77 (0.70–0.86)*
CT-scan	2429 (22.0)	5685 (38.8)	2.25 (2.13–2.38)*
Number of patients who had at least one consultation of another specialist on the ED n (%)	1617 (14.6)	6561 (44.8)	4.73 (4.44–5.03)*
Median length of stay (LOS) in ED in minutes [IQR]	200 [143–268]	234 [170–313]	*
ED revisit within 30 days n (%)	1483 (13.4)	2039 (13.9)	1.04 (0.97–1.12)
Destiny after ED visit^c^* n (%)*			
Admission	4368 (39.5)	7436 (50.8)	1.58 (1.50–1.66)*
Home	6415 (58.1)	6357 (43.4)	0.55 (0.53–0.58)*
Outpatient clinic	31 (0.3)	34 (0.2)	0.83 (0.51–1.35)
Institution	17 (0.2)	392 (2.7)	17.84 (10.97–29.01)*
Mortuary	47 (0.4)	53 (0.4)	0.85 (0.57–1.26)
Other^g^	92 (0.8)	260 (1.8)	2.15 (1.69–2.73)*
Median length of admission in days (IQR)^d^	2.7 [1.1–5.4]	4.7 [2.1–8.8]	*

*LOS* odds ratio, *ED* emergency department, *IQR* interquartile range, *OR* odds ratio

*p values were statistically significant and were calculated using χ2 tests, except for median length of stay on ED, which was calculated using Mann-Whitney U test

^a^Based on 25,102 ED presentations, due to 598 (0.8%) missings

^b^Based on 24,921 ED presentations, due to 779 (3.0%) missings

^c^Based on 25,502 ED presentations, due to 198 (0.8%) missings

^d^Based on 11,679 admissions, due to 125 (1.6%) missings

^e^Other transport: all transport modalities besides ambulance and private transport

^f^Acuity based on Manchester Triage Scale levels. Non-urgent: non-urgent and standard. Urgent: urgent, very urgent and immediate

^g^Other destiny after ED visit: all destiny’s after ED visits besides admission, home, outpatient clinic, institution and mortuary

### Impact of age and frailty on utility of acute medical care resources

**Fig. 3:**
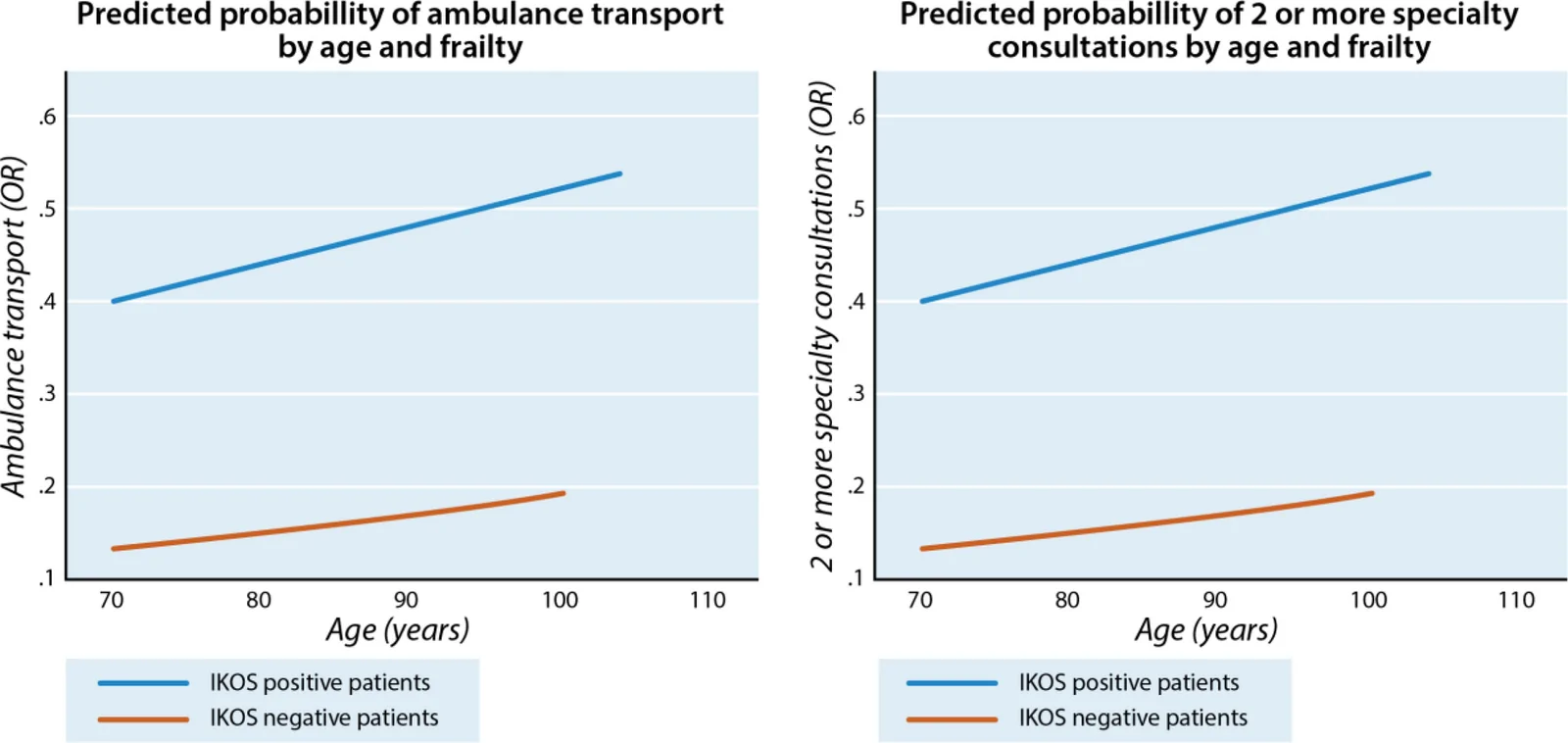
**a** Predicted probability (OR) of ambulance transport by age and frailty. Frail older patients (IKOS positive) have a higher change of being transported by ambulance to the emergency department. Age has also influence but less than frailty, **b** Predicted probability (OR) of 2 or more specialty consultations by age and frailty. Frail older patients (IKOS positive) have a higher change of having more consultations on the emergency department. Age has also influence, but frailty demonstrated a stronger association with the probability of specialty consultations on the emergency department

The extent to which age or frailty increases the likelihood of ambulance transport or a consultation in the ED of another specialty was determined. In a multivariable logistic regression analyses including both age and frailty (IKOS positive), both variables were independently associated with ambulance transport to the ED. For each additional year of age, the odds of ambulance transport increased by 4% (OR 1.04 (95% CI 1.04–1.04), *p* < 0.001). However, frailty had a substantially stronger association: frail older adults had more than twice the odds of ambulance transport compared to non-frail peers (adjusted OR 2.54 (95% CI 2.41–2.68), p < 0.001). These results suggest that frailty is a more powerful predictor of ambulance use than age among older ED patients. (Fig. 3a).

In a logistic regression model predicting involvement of multiple specialties during ED consultation, both age and frailty were independently associated with higher odds of multiple specialty involvement. Each additional year of age increased the odds by 1.7% (OR 1.02 (95% CI 1.01–1.02), *p* < 0.001). Frailty (IKOS positive) had a much stronger effect: frail patients had 4.45 times higher odds of involvement of two or more specialties compared to non-frail patients (adjusted OR 4.45 (95% CI 4.18–4.75), *p* < 0.001). The intercept (B = −3.052; OR 0.05) reflects the low baseline probability of multiple specialty involvement in non-frail patients at younger ages. (Fig. 3b).

## Discussion

Older adults have higher rates of acute medical care resource utilization, such as ambulance transport, diagnostics (laboratory and radiology), length of ED stay, hospital admission, and 30-day revisits compared to younger counterparts. This tendency is particularly pronounced in frail older adults and is independent of age in this group. Frailty is strongly associated with the need for ambulance services and the number of ED consultations. At the same time, these frail patients are not generally perceived as acutely ill by triage.

Previous literature on medical care resource utilization by older patients as compared to younger patients in the ED is scarce. Some studies describe demographic characteristics and high rates of ED visits by older patients [8–10]. Our findings align with these. Ukkonen et al., Latham et al., and Aminzadeh et al. observed that older patients often present with non-specific symptoms and diagnoses, leading to more expensive examinations and hospital admission with poorer outcomes. These studies did not consider frailty. Kallberg et al. [11] showed frailty prevalence of 57.3% in patients ≥ 70 years at the ED, consistent with our findings. They reported lower triage acuity, higher hospital admissions, and longer ED stays in frail compared to non-frail older patients. While they did not find high ambulance rates, they noted a higher risk ratio (1.18) for ambulance arrival when frail, consistent with our results.

Our findings are consistent with a flashmob study conducted by the European Taskforce on Geriatric Emergency Medicine (ETGEM). This multicenter, prospective observational study, in which standardized data were collected simultaneously over a short, predefined period, demonstrated that frailty assessment, rather than chronological age, is associated with worse outcomes. [12]. Higher Clinical Frailty Score (CFS) led to increased risk of hospital admission and death in the ED. Two large retrospective studies by Serina et al. and Wang et al. found nursing home residents use more ED resources than older individuals outside nursing homes; nursing home residency may serve as a frailty proxy representing the most vulnerable [13, 14].

Although frail older patients are transported more often by ambulance, they were less acuity triaged. One reason may be that the triage system is insufficient in older adults. Blomaard et al. demonstrated that triage via MTS alone poorly predicts mortality or admission risk for this group [15]. Over- and undertriage pose risks. The heterogeneity and atypical presentations in older patients complicate triage, but inappropriate triage alone does not fully explain lower triage levels despite higher ambulance use and acute care. Some frail patients may present to the ED without urgent medical indications. When older, frail patients experience acute deterioration, presentation at the ED and hospitalization is the current fallback option. In a study of Smeekes et al. they identified, other pivotal factors that drive older persons to an ED: functioning of the healthcare professional, availability of alternatives for the ED and patient frailty [16]. Nevertheless, when frail older adults do experience acute deterioration, timely recognition and appropriate triage are crucial. Our findings therefore underscore undertriage of frail older patients as a key clinical concern, with potential implications for patients safety and outcomes. Future research should focus on improving triage tools tailored to the older population and on identifying early markers for such deterioration.

The issue of overdiagnosis in the context of emergency medicine in frail and multimorbid older patients emerges as a significant consideration in our study. Intensive use of human and technological resources, may be linked to an increased risk of overuse, especially in critical medical situations. Our study shows frail patients undergo more diagnostics and consultations. Diagnostic uncertainty, atypical presentations, and disease specific guidelines may drive defensive medicine, leading physicians to order more tests than necessary as a precautionary measure, often without considering therapeutic consequences. Prior research showed increasing CT scan use in the ED over the years, with older age groups exhibiting the highest number of CT scans [17], aligning with our findings. It is uncertain, and unlikely, whether these rising rates lead to improved health outcomes for individuals [18]. Therefore, more use of ultrasound—which has lower potential harm—may be preferable for frail older patients. Yet ultrasound is underused, possibly due to low threshold for CT, ultrasound being time-consuming for radiologists, lack of routine lung ultrasound, and less attention to CT side effects [19]. Increasing use of handheld devices and point-of-care ultrasound by physicians may allow better management outside hospitals, with benefits [20, 21]. Lung ultrasound can discriminate pneumonia from other diagnoses in dyspnea better than X-ray and is more accurate in frail than non-frail patients [22].

Older patients often present with undifferentiated symptoms, multiple complex comorbidities, and geriatric syndromes like delirium, requiring multidisciplinary care [23]. EDs traditionally attend to younger patients with single-condition needs. Acute settings complicate systematic multidisciplinary meetings, causing sequential consultations and longer ED stays [24–26]. Staff shortages worsen this. Some presentations, such as pneumonia, may indicate pre-terminal events needing advance care planning beyond medical treatment [27]. A holistic approach considering patient wellbeing and goals is essential. Geriatric specialist involvement in the ED increases consultations; while often necessary, this may not be the only solution. Transmural strategies coordinating hospital and primary care may better address complex care needs across settings. Better insights in what specialists are consulted most by frail older adults could help to target on specific populations when it comes to prevention, transmural strategies and advance care planning. Future research is necessary to obtain more knowledge about these kind of health care utilization and trajectories.

Given that older patients often present with undifferentiated symptoms, multiple complex comorbidities, and geriatric syndromes such as delirium, a multidisciplinary approach is often required to address their diverse healthcare needs [23]. However, EDs have traditionally been designed to accommodate younger patients with more specialized, single-condition care needs. Due to the acute nature of the setting, it is challenging to schedule multidisciplinary meetings systematically, resulting in each discipline assessing the patient independently. This leads to multiple, often sequential, consultations, which are a significant factor contributing to longer lengths of stay in the ED [24–26]. A shortage of healthcare personnel exacerbates the issue, further extending the time patients spend in the ED. Furthermore, it is important to recognize that certain presentations, which may initially appear to involve a single condition, such as pneumonia, can in fact represent a pre-terminal event [27]. In such cases, medical treatment alone is insufficient; advance care planning becomes essential. This highlights the need for a holistic approach that considers not only clinical interventions but also the patient’s overall well-being, values, and goals of care. As our results clearly demonstrate, the involvement of a geriatric specialist here for at the ED, may contribute to an increase in consultations. While this may be inevitable given the complexity of care for frail older patients, it is worth considering whether this represents the most suitable or sole solution. Additional transmural strategies, involving coordinated care between hospital and primary care providers, may offer a more tailored approach to the complex care needs of frail older adults across care settings. This large retrospective study at a large teaching hospital offers novel insights on care utilization by older versus younger patients, focusing on frailty. No prior large studies have examined this impact. Some cardiac patients were excluded due to difficulty distinguishing elective from emergency admissions, limiting external validity for this subgroup. A practical screening tool rather than a validated one identified frailty, risking missed cases; however, there is no international consensus on screening tools [28]. The screening tool achieved high assessment rates. Mortality data, illness acuity, and revisit follow-up were lacking. Missing data were minimal.

This study utilized a large dataset from a large teaching hospital with an extensive ED. Though conducted retrospectively, it provides valuable novel insights in the utilization of care by older patients. Notably, there has been no previous research on such a large population focusing on medical care utilization in an older population compared to younger individuals, specifically examining the impact of frailty. It is important to acknowledge that a portion of patients, primarily those with cardiac issues, were excluded due to difficulties in distinguishing between elective admissions, like cardioversions, and true emergency cases. Consequently, the external validity for this specific sub group is limited.

The study employed a practical, feasible identification tool rather than a validated one, prompting concerns that some patients with indications of frailty might have been overlooked due to the lack of a more comprehensive screening process. Nonetheless, it is crucial to note that there is currently no international consensus on the appropriate screening tool to use [28]. On the other hand, the easy-to-use screening tool resulted in a high percentage of screened older patients.

Additionally, there is a lack of data regarding mortality and how acutely ill patients were, compounded by an absence of follow-up regarding representations in the first and last months of the study. We did not have access to detailed inpatient outcomes such as ICU admission or in-hospital mortality, as our dataset was limited to routinely collected ED data. Finally, there is some missing data, though this is relatively minimal.

## Conclusion and clinical implications

When compared with younger patients, older patients utilize more acute medical care resources within the ED and are more frequently admitted to the hospital, especially those who are frail. From a patient perspective, there are many reasons to question whether a hospital is the best place of care for frail older adults, from a system perspective this trend is not sustainable. Rather, diagnostic and treatment options should be considered in terms of their contribution to outcomes and sustainability, with respect to the individual needs and wishes of the patients. To reduce inappropriate hospital admissions and the incidence of nonbeneficial care for older people, shared decision making, advance care planning for acute episodes and/or future decompensation are solutions in combination with the use of alternatives to conventional hospitalization. Transmural team-based collaborations between hospital and primary care professionals may be one of these alternatives.

## Data Availability

The raw and processed data underlying this study are available from the corresponding author upon reasonable request. Data will be retained and made accessible for at least the period required by the data retention policies of our institution, in accordance with applicable regulations and guidelines.
